# *SgR1*, Encoding a Leucine-Rich Repeat Containing Receptor-like Protein, Is a Major Aphid (*Schizaphis graminum*) Resistance Gene in Sorghum

**DOI:** 10.3390/ijms26010019

**Published:** 2024-12-24

**Authors:** Hengyou Zhang, Liuling Yan, Yinghua Huang

**Affiliations:** 1 Department of Plant and Soil Sciences, Oklahoma State University, Stillwater, OK 74078, USAliuling.yan@okstate.edu (L.Y.); 2 USDA-ARS Plant Science Research Laboratory, 1301N, Western Rd, Stillwater, OK 74075, USA

**Keywords:** aphid, gene cloning, host plant resistance, insect resistance, LRR-RLP, sorghum

## Abstract

Greenbug, *Schizaphis graminum*, is one of the important cereal aphid pests of sorghum in the United States and other parts of the world. *Sorghum bicolor* variety PI 607900 carries the *Schizaphis graminum* resistance (*SgR1*) gene that underlies plant resistance to greenbug biotype I (GBI). Now, the *SgR1* has been determined as the major gene conferring greenbug resistance based on the strong association of its presence with the resistance phenotype in sorghum. In this study, we have successfully isolated the *SgR1* gene using a map-based cloning approach, and subsequent molecular characterization revealed it encodes a leucine-rich repeat containing receptor-like protein (LRR-RLP). According to DNA sequence analysis, the *SgR1* gene are conserved among GBI-resistance sorghum accessions but are variable within susceptible lines. Furthermore, an InDel (−965 nt) at its promoter region and a single-nucleotide polymorphism (SNP, 592 nt) in the CDS of the *SgR1* were detected and they are well conserved within resistant genotypes. When the *SgR1* gene was cloned and transferred into *Arabidopsis* plants, the *SgR1* was activated in the transgenic *Arabidopsis* plants in response to attack by green peach aphids according to the results of the histochemical assay, and GUS activity was detected in situ in spots around the vasculature of the leaf where the phloem is located, suggesting its biological function in those transgenic *Arabidopsis* plants. Overall, this study confirms that the *SgR1* gene coding for an LRR-RLP is the major resistance gene to greenbug, a destructive pest in sorghum and wheat. This represents the first greenbug resistance gene cloned so far and indicates that the simple-inherited GBI resistance gene can be used for sorghum improvement with genetic resistance to GBI via molecular breeding or cross-based conventional breeding technologies.

## 1. Introduction

Sorghum (*Sorghum bicolor* L. Moench) is the fifth most important cereal crop in the world in terms of acreage and production. Multiple uses of sorghum such as food, forage or biofuel feedstock, and its good adaptability in regions otherwise unfit for other cereals has made it an important staple crop that can ensure food security under the challenge of exponential population growth and climate change [1]. Greenbug, *Schizaphis graminum* (Rondani), is one of the key cereal aphid pests of sorghum in the Great Plains of the United States and many other parts of the world [2]. Greenbug aphids also cause damages on other major cereal crops, including wheat and barley, by sap-phloem sucking while injecting toxins into leaves through all growth stages [3,4], which results in various damage symptoms, including red spots or chlorosis on leaves, and failure in flowering and setting seeds. The estimated loss of sorghum by greenbug invasion is approximately $274 million annually [5]. With over 20 identified greenbug biotypes [6], greenbug Biotype I (GBI), identified and designated in 1990, has been the prevailing biotype that causes significant economic losses in sorghum-producing areas [3]. Most of the biotype E-resistant sorghum lines or hybrids were susceptible to GBI [3]. Three sorghum accessions, PI550607, PI550610 and PI607900 (also known as KS97), exhibited the highest level of resistance to both biotype E and I, and have been continuously used as resistant sources for sorghum improvement for resistance to greenbug [7,8].

It has been argued that new biotypes of aphids identified resulted from the pressure of the deployment of greenbug-resistant sorghum cultivars, or they shifted hosts from perennial, warm-season, non-cultivated grasses to new suitable over summering hosts for environmental adaption [9]. The winged virulent biotypes of *S. graminum*, such as GBI, migrate from the southern USA (e.g., south Texas) to northern areas (e.g., Nebraska) in late or early summer once a year, and have been dominant in these regions. Thus, it is necessary to control the expansion of GBI or to breed GBI-resistant cultivars by deployment of GBI-resistant cultivars or introduction of resistant genes into elite sorghum cultivars with the modern biotechnologies.

To better utilize resistant cultivars, genetic mapping was employed to identify the genomic regions that are associated with aphid resistance. Previous research efforts were made to identify the quantitative trait loci (QTLs) conferring tolerance to GBI. For example, at least three QTLs associated with resistance to greenbug biotype I were identified on chromosome SBI05, SBI06, and SBI07, respectively [10]. Agrama et al. [11] also identified another three resistant loci displaying GBI-specific resistance on chromosome SBI02, SBI05, and SBI09, and five non-specific resistant loci for GBI infestation by quantifying chlorophyll loss after GBI feeding. Likewise, Nagaraj et al. [12] used chlorophyll losses as indices to identify their GBI-resistant QTLs on sorghum chromosomes SBI01 and SBI04. In contrast to characterizing chlorophyll content in infested leaves, attempts were also performed by classifying the damage rate of sorghum leaves by greenbug, which also successfully located the causal genomic regions responsible for greenbug resistance. For instance, Wu and Huang [13] mapped a major QTL, *QSsgr-09-01*, and a minor QTL, *QSsgr-09-02*, on chromosome 09 of resistant sorghum PI550610, accounting for approximately 55–80%, and 1–6% of the phenotypic variation for GBI resistance, respectively. Similarly, Punnuri et al. [14] identified four overlapping major QTLs, *Qstsgr-sbi09i-iv*, conferring resistances to GBI on *S. bicolor* chromosome 09 by using a different resistant sorghum PI607900 instead of PI550610. *Qstsgr-sbi09i*-*iv* collectively accounted for 34–82% of phenotypic variance in GBI resistance [14]. These and other studies implicated that multiple chromosomal regions in sorghum are involved in sorghum resistance to GBI feeding, and SBI09 harbors at least one QTL. However, none of these loci were dissected in detail or cloned because of the tedious nature of positional cloning. The recent release of the *sorghum* reference genome sequence has facilitated the development of DNA markers and prediction of gene candidates that were co-localized with QTLs of interests [15,16].

Plants activate defenses or resistances to biotic stress by triggering a series of genes that are known as resistance (R) genes. The main class of R proteins consists of a leucine-rich repeats (LRR) domain(s), a nucleotide binding site (NBS), and a variable amino terminal domain, which are referred to by NBS-LRR R-proteins. NBS-LRR genes encode proteins that enable plants to recognize and defend against various biotic stresses, including pathogen or microbe, as well as a diversity of aphids in various plant species [17,18]. Zhu-Salzman et al. [19] identified an LRR-containing protein that showed significant accumulation of transcript in sorghum leaves within 48 h specifically after greenbug infestation. In addition to NBS-LRR proteins involved in resistance to biotic stress, R proteins can also be encoded by receptor-like proteins (RLP) genes that function as recognition and signaling in defensive reaction. But plant receptor-like proteins are far less well understood than NBS-LRR proteins. We have recently identified 308 R genes, including NBS-LRR, 175 genes encoding receptor-like kinases (RLKs), and 65 genes coding for receptor-like proteins (RLPs) in sorghum [*Sorghum bicolor* (L.) Moench] through genome-wide sequence analysis [20]. RLP genes encode R proteins that contain an extracellular LRR domain(s), a transmembrane (TM) domain, and an intracellular unknown domain. Several examples of RLP proteins as R proteins are also well documented. Tomato leaf mold pathogen *Cladosporium fulvum* (*Cf*) avirulence gene products can be recognized by a group of *Cf* genes-encoding RLP proteins in tomato, by which tomatoes trigger a resistance (hypersensitive) response in the presence of an extracellular cysteine protease Rcr3 [21,22]. In *Brassica napus*, blackleg resistant gene *LepR3* encoded an RLP protein that can interact with avirulence effector AvrLm1, which conferred *B. napus* resistance to the fungal pathogen *Leptosphaeria maculans* [23]. Typically, LRR motifs in these R proteins can form an α/β horseshoe shape that is involved in a protein–protein interaction in perception of *Avirulence* (*Avr*) gene products of pathogen and/or activate defense responses, both locally at the site of infection, as well as systemically throughout the plant. Additionally, mutations in conserved amino acids of a disease or insect resistant protein may result in distinct molecular activities leading to different aspects of defense [24]. The significant role of LRR-containing proteins, including RLPs in plant defense against biotic stress, indicates that this mode of R protein-involved plant defense is conserved and common in the plant kingdom.

It is speculated that products of these resistance genes play a role at intracellular or extracellular levels as “first guards” to interact with virulence proteins of phytotoxic salivary components secreted by aphids, and this follows a gene-for-gene hypothesis [25]. Many RLP-type R proteins were identified in various plant species, whereas RLPs with two TM domains have not been reported as R proteins. To date, more than ten greenbug resistance genes have been reported in sorghum and wheat, and the mode of action of each needs to be further determined. Thus, the objective of this study was to isolate and characterize the genes conferring greenbug resistance in sorghum. To achieve this goal, we performed map-based cloning of the *SgR1* locus on *S. bicolor* chromosome 09, investigated its expression pattern under greenbug infestation, and studied the allelic variation of the *SgR1* in various sorghum accessions. Here, we report the successful cloning of the causal genes responsible for GBI resistance in sorghum and molecular characterization of the *SgR1* gene. These important findings not only shed light on further investigations to dissect the mechanism of plant-aphid interactions but contribute to a useful tool for sorghum improvement with resistance to greenbug aphids.

## 2. Results

### 2.1. Determination of SgR1 Locus

**Figure 1:**
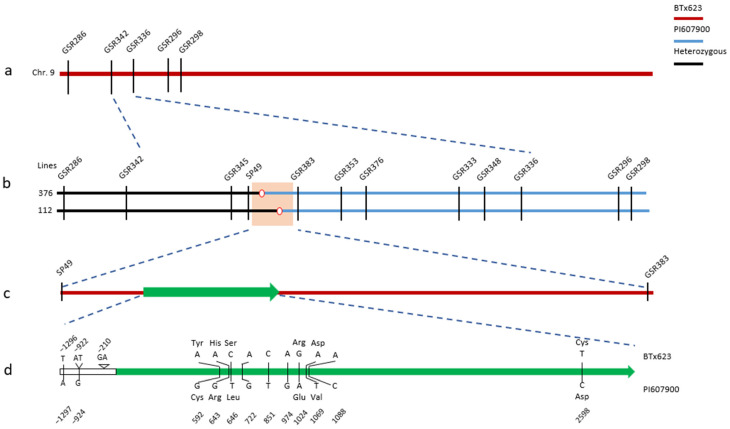
High-resolution genetic map of the *SgR1* on chromosome 9 of *S. bicolor*. (**a**) Genetic linkage map after QTL validation showing the position of the *SgR1* flanked by GSR286 and GSR298 and narrowed into GSR342 and GSR336 with additional markers. (**b**) Plants (lines 376 and 112) with critical crossovers flanking the *SgR1.* The *SgR1* was delimited into a region flanked with dCAPS marker SP49 and marker GSR383. The white and red circles indicate the point where the chromosomes broke and then reattached to another chromosome. (**c**) A single putative gene in the candidate region predicted by sorghum reference genome (v.2.1), FGENESH and Gramene. Solid arrows represent exon and transcription orientation. (**d**) Sequence comparison of the SgR1 promoter regions and open reading frame between the resistant parent PI607900 and the susceptible BTx623. White bar represents promoter region. Solid arrow represents exon and transcription orientation. Nucleotide variants and their respective position in the *SgR1* from each genotype were indicated above or below the gene. Amino acid change was placed on corresponding side of nucleotide-variants.

In our previous study, four QTLs conferring sorghum resistance to GBI were mapped on a coinciding genomic region flanked by DNA markers Starssbem286 (GSR286)–Starssbnm102 (GSR102) on sorghum chromosome 9, accounting for 64.7–82.4% of the phenotypic variation [14]. The overlapped genomic region from GSR286 to GSR298 was targeted for QTL dissection here (Figure S1). First, we validated this region using the offspring of the recombinants (Lines 277, 275, 231, 218, and 157) that had been identified in the F_2_ population, leading to the confirmation of which recombinant lines carry the DNA fragment associated with aphid resistance, including RILs 277 and 275, thus others were eliminated. After analysis of genotyping with markers GSR 286 and GSR298, and phenotyping by GBI infestation (Figure S2), genes underlying the QTL were targeted between markers GSR 286 and GSR298. These DNA markers proved to be effective and reliable in marker-assisted selection. Furthermore, this QTL showed a 3:1 segregation (χ^2^ = 0.83, *p* > 0.05) in L277 progenies in response to GBI feeding, suggesting that sorghum resistance to GBI infestation was controlled by a single gene. We designated this gene as *Schizaphis graminum Resistance 1* (*SgR1*). By developing and analyzing additional markers GSR342, GSR345, GSR333, GSR336, and GSR348 within the *SgR1* region, we further identified the crossover events that occurred for the five lines (Figure S1). The results of genotyping and phenotyping of lines L231, L275, and L157 narrowed the *SgR1* region into a 125-kb interval flanked by GSR342 and GSR348 (Figure 1a,b).

To map-based clone the *SgR1*, recombinants with crossovers in the 125-kb interval were analyzed. After screening around 4000 individuals with greenbug biotype I (GBI), three *SgR1*-carrying F_2:7_ RIL lines (303, 313 and 338) exhibited segregation. These three lines also showed heterozygosity at both markers, GSR342 and GSR336. By large-scale genotyping of F_2:8_ plants from self-crossed F_2:7_ plants carrying a heterozygous *SgR1* segment with newly developed markers, a total of 49 recombinants carrying a recombination point between the two markers (GSR342 and GSR336) were identified. Of these 49 recombinants, two key recombinants (L112 and L376) were selected for further analysis (Figure 1b). Genotypes of the *SgR1* in each of the recombinants were determined by evaluating the progeny phenotype under greenbug biotype I infestation. As shown in Figure 1 and Figure S2, the *SgR1* locus is homozygous in recombinant L376, while heterozygous in recombinant L112. These results, in combination with the phenotypic patterns of their progenies, implied that the *SgR1* gene was delimited to a genomic region between markers SP49 and GSR383. As shown in Figure 1c, this delimited region is physically 12-kb on the genome of BTx623 and PI607900 by referring to reference genomic sequence data and DNA sequencing, respectively.

### 2.2. SgR1 Gene Encodes a Receptor-like Protein (RLP)

Further analysis of this 12-kb region by available sorghum annotations, FGENESH (http://www.softberry.com/, accessed on 19 November 2024) and Gramene (http://gramene.org, accessed on 19 November 2024), predicted a single gene (Sobic.009G012900) (Figure 1c) which was designated as dominant *Schizaphis graminum Resistance gene 1* (*SgR1*) in the resistant PI607900 and the recessive *Sgr1* in the susceptible BTx623 (Figure 1c). Both alleles possess an intact coding sequence of 2961 bp in length without introns by comparing genomic and cDNA sequences through sequencing. In addition, a sequence comparison revealed that a total of ten single nucleotide polymorphisms (SNPs) were detected in their coding sequence (Figure 1d), of which six SNPs result in amino acid changes between PI607900 and BTx623. Other than the differences in the coding region, three SNPs or indels (2 bp) and one SNP were also detected between the *SgR1* and the *Sgr1* in the promoter regions (1500 bp upstream of the start codon) and the 3′ regions, respectively (see the upstream of Figure 1d).

**Figure 2:**
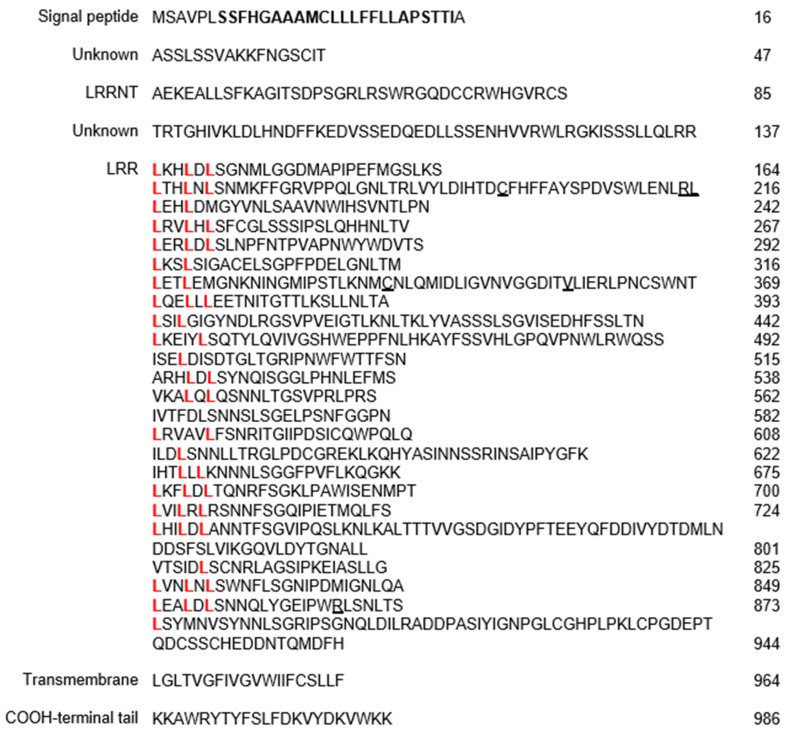
Predicted amino acid sequence of the *SgR1* gene product. The deduced protein domains are indicated as a signal peptide and transmembrane domain (bold), unknown function, LRRNT, unknown function, LRR domains with leucine (L in red), and a COOH-terminal tail. Nucleotide variations resulting in the respective amino acid changes between parental lines are underlined.

The open reading frame (ORF) of the *SgR1* encodes a 986-aa protein Pfam and SMART (http://smart.embl-heidelberg.de/, accessed on 19 November 2024) analysis revealed that the SgR1 protein is an RLP that contains 24 extracellular LRR domains (Figure 2, indicating as L in red) at the C terminal region, an N-terminal short peptide ranging from 1–26 amino acids, and a TM domain localized at C- and N-terminal regions, respectively (Figure 2). This domain arrangement is similar and has been detected in other RLP R proteins, such as Cf proteins, RLM2, and LepR3 [21,26,27,28]. For example, the protein SgR1 possesses a 30-amino acid (aa) signal peptide overlapping with a TM region (residues 7–29) at the N-terminal followed by an extensive extracellular LRR region (residues 48–944), a transmembrane motif (residues 945–964) close to the N-terminal and a 21-aa cytoplasmic short peptide at the C-terminal (Figure 2).

### 2.3. Allelic Variations of SgR1 in Sorghum Varieties

**Figure 3:**
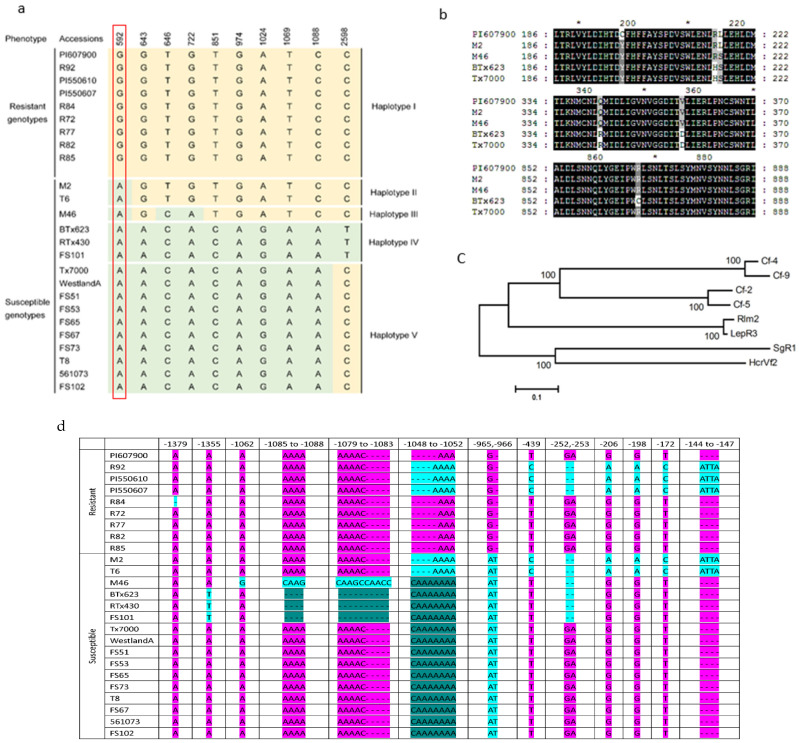
(**a**) SNP analyses and multiple nucleotide sequence alignment of the SgR1 alleles in different lines, in which the conserved bases are highlighted in orange or light green and key variations were framed by a red rectangle and same nucleotide variants at a single position are highlighted with the same highlight color. (**b**) Shows the partially deduced proteins with variations. (**c**) An unrooted phylogenetic tree constructed with the SgR1 and other RLPs proteins using MEGA software (version version 4.0.) (https://www.megasoftware.net/, accessed on 19 November 2024), and the numbers at nodes represent bootstrap values per 1000 replicates as determined by NJ method. Protein accession numbers used in the tree construction are as follows: Cf-4 (CAA05268, *Solanum habrochaites*), Cf-2 (AAC15779, *Solanum pimpinellifolium*), Cf-9 (A55173, *Lycopersicon esculentum*), HcrVf2 (CAC40826, *Malus floribunda*), LepR3 (AGC13587, *Brassica napus*) Cf-5 (AAC78591, *Solanum lycopersicum*), *Rlm2* (KM097068, *Brassica napus*). (**d**) Shows sequence variations in the promoter regions of various genetic lines. Different colors in the figure indicate those in different subgroups.

To establish a link between the *SgR1* alleles and sorghum resistance to GBI, we analyzed 22 sorghum lines (8 resistant; 14 susceptible) in addition to the parental lines for their response to GBI infestation and genotype of the *SgR1*. Two GBI resistant genotypes (PI550607 and PI550610), a resistant panel and GBI-susceptible sorghum accessions RTx430, Tx7000 and Westland A were previously identified [10,13,29,30,31]. The full length of the *SgR1* as well as a 1.4-kb promoter region were fully sequenced in these 24 sorghum genotypes. Comparison of predicted coding sequences (CDS) and proteins revealed that all the resistant and susceptible accessions possess the same sequence in length, with 2961-bp CDS encoding 986 amino acids. As shown in Figure 3a, all the tested genotypes exhibited SNP variation in the coding region of the *SgR1* that was observed between PI607900 and BTx623. Five different *SgR1* haplotypes were identified, in which there are variations in those partially deduced protein sequences (Figure 3b). Sorghum lines with haplotype I carry the resistant alleles of *SgR1* that show resistance to GBI, and these SNPs in haplotype I are conserved (Figure 3a). However, varied genotypes designated as Haplotype II to V were observed at these locations in the susceptible group. Among the 10 SNPs identified, sorghum resistance to GBI infestation can be distinguished at position 592 of the *SgR1* alleles coding region, which resulted in amino acid change of Try (C) in PI607900 and Cys (Y) in BTx623 and other susceptible genotypes. The PI607900 haplotype exhibited a strong correlation with the resistance phenotype.

**Figure 4:**
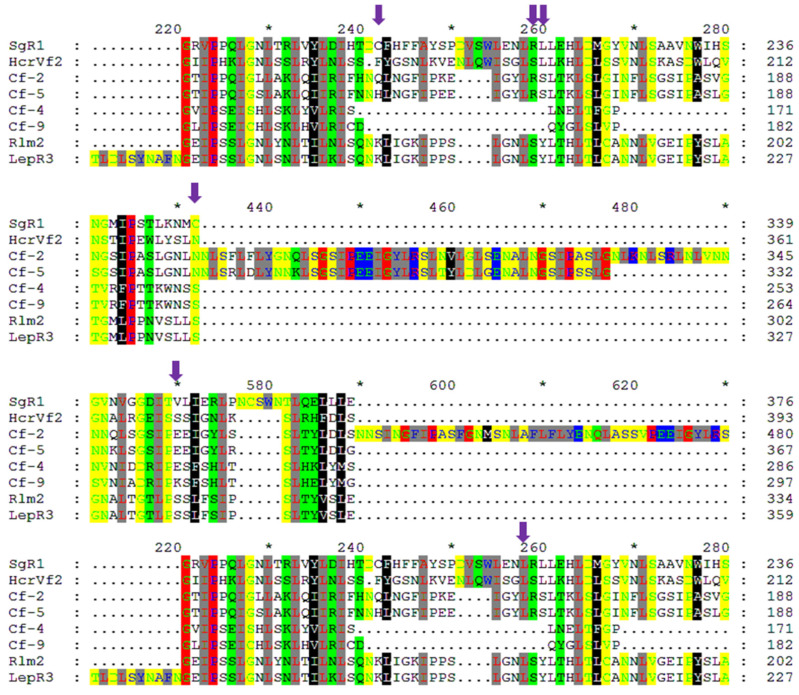
Multi-sequence alignment of the SgR1 with other published RLP R proteins. Purple arrows indicate the amino acid variants in the SgR1 proteins. Amino acids in a column that have similar properties are highlighted with the same colors.

Several amino acid variants in PI607900 are conserved in some plant species. To determine if the amino acid in PI607900 is conserved, we compared the deduced SgR1 with other known RLP R proteins through multiple-sequence alignment. As shown in Figure 4, in addition to highly enriched Leucine (L), several other amino acids are highly conserved, such as proline (P), asparagine (N), glycine (G), and serine (S). We also noticed that several amino acids exhibit similarity with other RLP proteins. For example, Arginine (R) at position 259 is conserved in two tomato Cf protein members Cf-2 and Cf-5, and its adjunct amino acid Leucine (L) at position 260 is also identical to that in HcrVf2. These results suggest that these conserved amino acids might function for specificity of resistant *LRR-RLP* alleles. On the other hand, there are a few unique amino acid variants in the SgR1 protein as indicated by purple arrows (Figure 4), possibly suggesting their specific role in resistance to aphids.

In addition to the conserved SNPs found in the coding region of SgR1, high variability was observed in the 1.4-kb promoter region of *SgR1* in resistant accessions (Figure 3c). In the 1.4-kb promoter region, we identified a total of 13 sequence variations comprising six indels and seven SNPs. All the identified variations showed no correlation with sorghum resistance in the 24 selected genotypes except indel G/AT at −965/966 bp upstream of the start codon of *SgR1*. More specifically, at this location, all the resistant sorghum accessions possess nucleotide G, whereas the AT were identified in all 16 of the selected susceptible sorghum accessions. Thus, the genotyping results support that the SgR1 is a putative causal gene for GBI resistance.

We also investigated the cis-regulatory DNA elements at the promoter region of the *SgR1* alleles. We found some sequence portion in the promoter region are the targets of well-studied transcription factors (TFs); for example, MYB binding sites TAACTG at −72, MYC binding sites CANNTG at −29, and WRKY binding sites TGAC, −146 and −173 upstream of the start codons in the *SgR1* alleles. These TFs are involved in diverse plant responses to abiotic and biotic stresses [32,33,34,35,36]. In addition, we also detected a cis-regulatory DNA element LTRECOREATCOR15 (CCGAC) at the G/AT variation locus in all the resistant genotypes. These DNA variants confer cold, drought- and ABA-regulated gene expression in various plant species [37,38]. However, no cis-regulatory DNA elements were predicted in other DNA variants of promotors.

### 2.4. Expression Analysis

**Figure 5:**
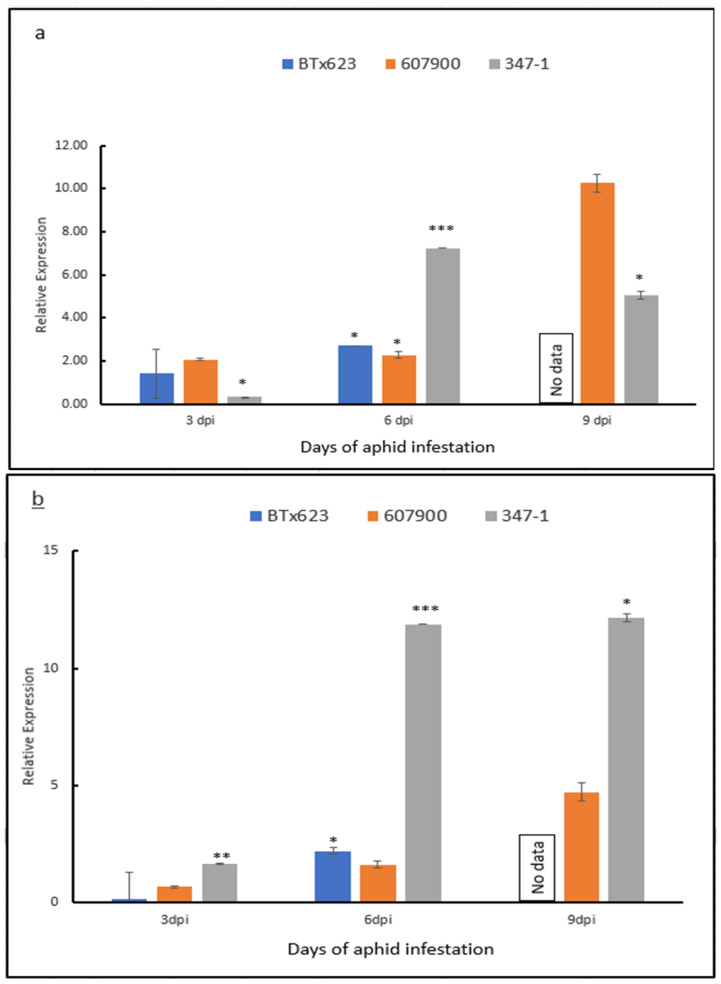
Analysis of the *SgR1* in response to GBI infestation using *q*RT-PCR, showing the relative expression of the gene in three genotypes using the 2^−ΔΔCt^ method. (**a**), relative expression of the *SgR1* gene in three lines (BTx623, PI607900, and 347-1 at 3, 6, and 9 dpi in response to GBI infestation vs. none-infested control; and (**b**) relative expression of the *SgR1* gene in three lines (BTx623, PI607900, and 347-1 at 3, 6, and 9 dpi compared to 0 dpi. No data means no expression data because seedlings in susceptible line were severely damaged at 9 dpi. Error bars represent standard error among replicates (n = 3) and the asterisks represent statistically significant changes between the controls and aphid infested samples as determined using Student’s *t*-test, * *p* <  0.05, ** *p* <  0.01, *** *p* <  0.001 while the bars without asterisk are non-significant (*p* > 0.05).

We performed gene expression studies to investigate the expression profile of the *SgR1* over a time course by qRT-PCR. As shown in Figure 5, the *SgR1* was induced by GBI infection in both resistant PI607900 and 347-1 (resistant recombinant derived from PI607900), and susceptible BTx623. Specially, the *SgR1* kept a relatively lower expression level in all genotypes when they were free of GBI attack, whereas a significant difference was observed between resistant and susceptible genotypes at 3 days post infestation (dpi), where the expression levels of the *SgR1* in PI607900 and 347-1 were significantly higher than in BTx623. Although the *SgR1* showed upregulation in all lines following GBI infestation, upregulation in resistant genotypes was much higher than in the susceptible line. The *SgR1* showed a significant difference between PI607900 and BT623 at both 3 and 6 dpi, the expression level in the recombinant (347-1) was much higher. Nevertheless, the quantitative RT-PCR results showed that the *SgR1* is inducible by GBI infection, implying that the SgR1 recognized the GBI attack and its role in plant defense.

**Figure 6:**
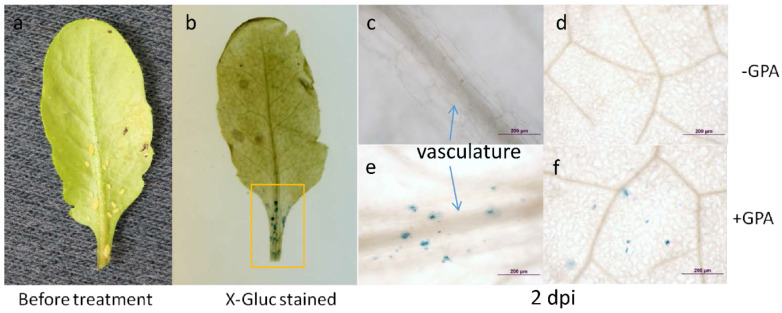
SgR1p::GUS expression in GPA-infested and non-infested *Arabidopsis* leaves. The leaf with colonized GPA aphids at 2 dpi (**a**) and showing histochemical staining (see blue spots in the yellow square) for GUS activity in the leaf (**b**). GUS activity was not detectable on the non-infested leaves (**c**,**d**). Blue colorations showing SgR1p::GUS expression at both vasculatures (**e**) and other tissues (**f**) of infested leaves. Scaled bars 200 µm.

With intention to determine whether the *SgR1* is functional in the model plant Arabidopsis, it was introduced into the Arabidopsis genome through transformation. In this experiment, we cloned approximately 1.9-kb of the *SgR1* promoter region (SgR1p) into the upstream of the β-glucuronidase gene in the expression vector pCAMBIA1302 to generate transgenic *Arabidopsis* plants. Because *Arabidopsis* is not the host of GBI, *Arabidopsis* plants were challenged by GPA. The GPA-infested and non-infested leaves of positive *Arabidopsis* expressing *SgR1p*::*GUS* were used for inspection. Following introduction of GPA to the plant pot, we observed a number of aphids underneath the leaves at 2 dpi (Figure 6a). Then, we tested heterologous expression of the *SgR1* gene in *Arabidopsis* plant infested by green peach aphids (GPA), so evidently the *SgR1* gene rapidly responded to GPA attack in Arabidopsis according to the results from histochemical assay (Figure 6).

As expected, blue coloration indicative of GUS activity was expressed in situ spots around the vasculature of the leaf (Figure 6b). Zooming into the X-Gluc stained region, the blue spots were mostly located on vasculature or rarely found at non-vascular cells (Figure 6c,e). By comparison, no GUS activity was observed on non-infested *Arabidopsis* leaves (Figure 6d,f). These results suggested that the *SgR1* was inducible by aphid infestation and triggered and functioned in the vascular tissues to defend against aphid attack.

### 2.5. Phylogenetics Analysis of SgR1 Alleles

To understand the relationship between the *SgR1* and related alleles the identified RLPs showing resistance to various biotic stresses were used to construct a phylogenetic tree using the SgR1 with 18 proteins from the published literature and the NCBI database. As shown in the tree, the SgR1 was clustered with RLPs from various monocotyledons, and evolved closely to RLP12 from wheat. Surprisingly, the SgR1 evolved further with Cf family proteins that were the first reported RLPs functioning resistance to biotic stressors, and the pathogen resistant protein LepR3 identified lately [21,22,27]. Although the other three are LRR-containing R proteins, they outgroup the RLP subgroups. An unrooted phylogenetic tree shows the relationship between the SgR1 and the other seven RLPs proteins (Figure 3c).

In the reference genome of BTx623, our genome-wide analysis led to identification of 56 RLP genes including the *SgR1*. A phylogenetic tree was constructed using the full length of deduced amino acids of *SbRLP*s. The tree was split into four groups (Figure S3). The SgR1 groups with 28 SbRLPs into the largest group. In addition, we identified six pairs of duplicated RLPs that were located at *S. bicolor* chromosomes 01, 03, 04, and 08, whereas no duplicated pairs of RLPs were found for the SgR1 (Figure S3).

## 3. Discussion

The GBI resistance genes were mapped on several chromosomes of *S. bicolor* using different resistant genotypes as objects, and two QTLs were mapped at a coinciding region on chromosome 9 [13,14,30]. By analogy to the mechanism of R protein-mediated plant-pathogen interactions [39], perhaps plant defenses against aphids might also be manipulated by R proteins. In the present study, we report the cloning of QTL *Qstsgrsbi09*, the first *S. bicolor* LRR-RLP conferring resistance to *Schizaphis graminum*, which provides a gene resource available for the dissection of sorghum–greenbug interaction. Although the SgR1 share only 33.74% and 27.01% identity with apple HcrVf2 and tomato RLP Cf-9, respectively, they share a similar organization of the main domains. A horse-shoe structure of the SgR1 by 3-dimentional structure prediction is in accordance with the hypothesis demonstrating the putative function pattern of LRR-containing R proteins [40]. The eLRR region in the SgR1 and other aphid resistant proteins, for example, *Mi* from tomato [17], *BPH14* from wild rice [41], and *BPH26* from rice [42], and the ribonuclease inhibitor (RI) are key and necessary domains responsible for plant resistance to aphid attack. The similarity in primary protein structure of these published LRR-RLPs implicated the gene-encoding LRR-RLP here is the putative *SgR1* [20].

The host-plant resistant gene, SgR1, is conserved in GBI-resistant sorghum varieties. The *SgR1* and alleles conferring resistance to GBI from PI607900, PI550610, and PI550607 were widely used as resistant sources in breeding GBI-resistant sorghum [7,43]. In contrast to the diverse alleles of LepR3/Rlm2 in *Brassica napus* varieties [28], the sequence of the SgR1 is conserved within limited resistant sorghum varieties, suggesting that the *SgR1* possibly experiences little selection pressure from attack by greenbug biotype I. Of six conserved amino acids between the resistant SgR1 and other sgr1 proteins, five were in the ribonuclease inhibitor (RI) domain, which is a functional core contributor to its defense [40]. In addition, the LRR region close to the N-terminal can determine the functional specificity of the plant RLPs [44]. Cf-9 activity was severely affected by the substitution of conserved Trp (W) in the motifs close to the N termini [45]. An aphid-related study also demonstrated that substitution of certain amino acids of the PAD4 protein altered its function specificity in GPA resistance [26]. Although amino acid variations occurring at the N-terminal of sorghum homologs SgR1 and conservation in the second and third amino acid between the SgR1 and other published LRR-RLPs [21,40] suggests this region plays an important role in determining the specificity of the SgR1; however, additional protein function testing is needed.

The results of this project demonstrated that an approach involving recombinant inbred lines and marker-assisted selection was quite robust and reliable. A panel of recombinant inbred lines (RILs) were created through a cross between susceptible and resistant parents (BTx623 and PI607900), then a large number of offspring were evaluated and quickly narrowed down a few targeted RILs based on phenotypic variation in GBI resistance and correlation with the marker genotype (Figures S1 and S2). Evidently, L277 was selected as a candidate line for gene cloning experiment, leading to the successful cloning of the *SgR1* gene. Our success proves the recombinant inbred lines and marker-assisted selection as powerful tools for QTL identification and gene cloning.

Analysis of cis-acting elements in the SgR1 promoter region and their putative transcription factors may provide a preliminary understanding of the regulatory system in plant–greenbug interaction. Several WRKY and MYB TFs involved in the crosstalk of ABA signaling pathways during biotic stress responses have been characterized in various plant species [36,46,47,48]. In addition, ABA signaling and biotic stress signaling share many common components, and different ABA levels exhibit different plant resistance towards pathogens [49]. The cis-acting elements predicted in the promoter region implicated the involvement of WRKY or MYB TFs in sorghum–greenbug interaction [36]. It is noted there was a strong correlation between the resistant variant at the G/AT locus in the promoter and the first amino-acid variants of the SgR1 with sorghum GBI resistance. The LTRECOREATCOR15 is thought to play a key role in transcribing the *SgR1* to trigger the defense but needs further investigation. Nevertheless, these findings provide clues about the role of ABA signaling elements or ABA-involved callose deposition at feeding sites during sorghum–aphid interactions [50]. These variations in the promoter region may contribute to the different levels of resistance in these genotypes. However, since the *SgR1* alleles in both parental lines are inducible with similar expression patterns by GBI infestation (Figure 5), it suggests the cis-acting element appears not to be a requirement for *SgR1* transcription. This finding also needs further determination.

Different species of aphids shared some effector proteins that can trigger plant defense. Nicholson and Puterka [51] detected several proteins that are common in four differentially virulent greenbug biotypes by analyzing their exuded saliva. Since both sap-sucking greenbug aphid and green peach aphid belong to Aphididae, we boldly hypothesize that both aphid species may evolve closely to some extent in their feeding system, secreting common or similar virulent proteins. Our histochemical assay results demonstrate that certain salivary proteins in GPA saliva trigger components that can bind the *SgR1p* attempting to activate the *Arabidopsis* defense system. These results also suggest the common virulent proteins secreted by both GBI and GPA are conserved to some extent in the defense system to aphid resistance in *Arabidopsis* and sorghum. Moreover, it remains to be determined if the *SgR1* can be transcribed by aphid infestation and functions in vasculatures where aphids have settled down for feeding. Collectively, these results suggest that the *SgR1* cloned in the present study is the major gene conferring GBI resistance in sorghum.

It is a widely accepted idea that sorghum originated in Africa, and was extensively introduced and domesticated to Arabia, India, China, and the United States of America [52]. For the three selected GBI-resistant sorghum accessions, PI607900 was developed from a South African germplasm, and sorghum accessions PI550610 and PI550607 were collected in Syria and China, respectively. Although they are genetically distinct, it is reasonable that these genotypes possess an identical *SgR1* conferring GBI resistance. During germplasm introduction and artificial selection via conventional breeding, the *SgR1* has evolved and inherited through selection for yield, quality, or other traits of interests, which was followed by accessions in migration to sorghum-growing areas. The *SgR1* in different genetic backgrounds might lead to different levels of resistance to GBI attack, as described previously [13,14]. Greenbug on sorghum has been documented back to 1868 [53] (Webster and Phillips, 1912) and was a serious pest of sorghum in the 1960s [2]. Sequence analysis is also consistent with the fact that the *SgR1* appears to experience low selection pressure on sorghum until an outbreak of GBI that was first identified in the 1990s [3]. More *S. bicolor* accessions with different levels of resistant responses to GBI infestation from different geographical areas are needed for the ecological and evolutionary understanding of the sorghum–greenbug interaction.

In summary, the *SgR1* that confers resistance to GBI was successfully cloned for the first time. Molecular characterization demonstrates that the *SgR1* encodes a leucine-rich repeat containing receptor-like protein (LRR-RLP), which is also well known as the main part of most disease resistance genes in plants. Structure analysis revealed that the *SgR1* possesses all key domains and characteristics of R genes for proper regulatory function for host plant resistance to phloem-feeding aphids. This study also confirms that the SgR1 was upregulated in response to GBI infestation and was expressed in transgenic Arabidopsis and became active in response to attack by green peach aphids according to the gene expression results, suggesting its role in plant defense against aphids. Overall, this study confirms that the *SgR1* gene coding for an LRR-RLP is the major greenbug resistance gene in sorghum and indicates that the simple-inherited GBI resistance can be used for genetic improvement of sorghum crops with genetic resistance to GBI via cross-based conventional breeding or molecular breeding technologies.

## 4. Materials and Methods

### 4.1. RIL Population Development

A population of 96 RILs (F_2:7_) were derived from a cross between two sorghum varieties BTx623 and PI607900, developed in our lab, and was used in this study. Susceptible sorghum BTx623 was used to produce the reference genome sequence [15]. PI607900 provides superior resistance to GBI than other germplasm sources previously available [43]. The early generation F_2:3_ containing 371 individuals of this population had been used previously for linkage map construction and QTL analysis for sorghum resistance to GBI [14]. Four major GBI resistant QTLs, *Qstsgr-sbi09i-iv*, were identified at a region flanked by GSR286 and GSR296 on SBI09 (chromosome 09) with the foliage damage rate as a trait at four time points post infestation [14]. This population subsequently advanced to a population consisting of 96 F_2:6_ recombinant inbred lines (RILs) by the single seed descent method. The F_3_ and F_4_ populations were grown in the greenhouse at the USDA-ARS in the winter of 2010–2011. Subsequent phenotyping by GBI infestation of the F_5_ population was also performed in the USDA-ARS greenhouse.

Seeds of the lines with segregating resistance to GBI infestation and with a heterozygous *SgR1* region on *S. bicolor* chromosome 9 were bulked and planted for recombinant screening using QTL-flanking markers GSR336 and GSR342. Identified recombinants were self-pollinated in the beds of the greenhouse to collect the seeds and confirm the phenotype of the progeny for fine-scaled linkage mapping.

### 4.2. Aphid Rearing, Infestation, and Phenotyping

Greenbug biotype I (GBI), *Schizaphis graminum* (Rondani) has been maintained in our lab, USDA-ARS, Stillwater, Oklahoma for several decades. Rearing of GBI on susceptible barley seedlings and infestation on sorghum seedlings were followed as previously described [30]. GBI genotyping was conducted in the USDA-ARS greenhouse under the controlled growth conditions as previously described [14]. The plants for GBI phenotyping were planted in flats filled with potting mix and regularly watered. To determine the resistance segregation of 96 RILs, five seeds per line and three replications were used for genotyping. The 10-day-old sorghum seedlings were used for GBI infestation. A discrete scale that ranged from one to six was used to represent foliage damage at the level of ≤20%, 20–40%, 40–60%, 60–80%, ≥80%, and dead, respectively. Seedlings of BTx623 and PI607900 were used for susceptible and resistant control, respectively. GBI rearing, infestation, and growth of sorghum plants were conducted in the greenhouse under the conditions of 28 ± 2 °C with 14 L/10 D photoperiod according to the previous report [54].

Green peach aphids (GPA) (*Myzus persicae* Sülzer) were reared on an equal mix of radish (*Raphanus sativus* ‘Early Scarlet Globe’) and mustard (*Brassica juncea* ‘Florida Broadleaf’) in a growth chamber programmed for a 14 L/10 D photoperiod at 22 °C as described by [24]. Twenty GPA adults were gently put on leaves of the 4-week-old, caged *Arabidopsis* plants. At least three individuals were infested and used for GUS activity determination.

### 4.3. Isolation of the SgR1 Through the Map-Based Cloning

Genomic DNA was extracted from dried leaves using modified cetyltrimethylammonium ammonium bromide (CTAB) method [55] (Murray and Thompson 1980). The single sequence repeat (SSR) marker was predicted based on the *S. bicolor* reference genome of BTx623. Insertion-deletion (Indel) and single nucleotide polymorphism (SNP) markers were developed by resequencing the *SgR1* genomic regions of PI 607900 and comparing them with BTx623. SNPs were developed into dCAPS markers. A candidate DNA fragment was obtained from the QTL region through the map-based cloning method using the primers designed based on DNA sequences in the QTL region (Supplementary Table S1). The forward primer of each SSR or Indel was tailed with M13 forward primer sequence (5′-CACGACGTTGTAAAACGACG-3′) before the 5′ end of the sequence. A concentration of 10 ng/μL of genomic DNA was used as a template, and PCR reactions were programmed with an initial denaturation of 94 °C for 5 min, 14 cycles at 96 °C for 30 s, 58 °C for 1 min, 72 °C for 30 s, then 28 cycles at 96 °C for 30 s, 55 °C for 1 min, 72 °C for 30 s, and followed by extension at 72 °C. PCR products that were amplified with M13 forward primer labeled with either 700 or 800 nm florescent dye were separated in 6.5% polyacrylamide gels in a LI-COR 4300 DNA Analyzer (Li-Cor Inc., Lincoln, NE, USA). PCR products of dCAPS primer SP49 (Table S1) were digested by restriction enzyme, and then separated on 3% (*w*/*v*) agarose gels and visualized with ethidium bromide.

### 4.4. RNA Extraction and Gene Expression Analysis

Total RNA was extracted from the leaves of PI607900 and BTx623 with TRIzol (Life technologies, Carlsbad, CA, USA). Genomic DNA was removed using DNase I (New England Biolabs, Ipswich, MA, USA). The first strand cDNA was synthesized using QIAGEN RT-PCR Kit (Qiagen, Germantown, MD, USA) according to the manufacturer’s instructions. All quantitative real-time polymerase chain reaction (*q*RT-PCR) experiments were performed using an iQ5 system (Bio-Rad, Hercules, CA, USA), using iQ5 SYBR Green Supermix (Bio-Rad, USA) as directed by the manufacturer. Specific primers (SgR1_qPCR) for *q*RT-PCR are shown in Supplementary Table S1. Three biological and two technical replications were used in this analysis.

### 4.5. Sequence Comparisons of SgR1 Among Sorghum Varieties

The 1.4-kb promoter region and exon region of the SgR1 alleles from various *S. bicolor* accessions were PCR amplified with specific primers (SgR1_promoter and SgR1_CDS, Table S1) using genomic DNA as a template. Those PCR products were gel purified using a QIAquick Gel Extraction Kit (Qiagen, USA) and sequenced at Oklahoma State University’s Core Facility for sequence comparison. cDNA sequences from parents were also sequenced for comparison. Deduced amino acids of the *SgR1* alleles were also compared. The cis-regulatory DNA elements in the promoter region were predicted in database PLACE (http://www.dna.affrc.go.jp/PLACE/, accessed on 19 November 2024).

### 4.6. Phylogenetic Analysis

The sequence alignment was conducted using software Clustal X version 2.0 [23] and visualized by GeneDoc (http://www.nrbsc.org/gfx/genedoc/ebinet.htm, accessed on 19 November 2024). The phylogenetic analysis was conducted using MEGA4 program. To retrieve all of the LRR-RLPs from the reference genome of *S. bicolor* (version 2.1) that downloaded from the database (http://phytozome.jgi.doe.gov/pz/portal.html, accessed on 19 November 2024), we used 11 different Hidden Markov Model (HMM) profiles (LRR1 to 9, LRRNT1to 2) of the LRR domain downloaded from the Pfam database as inquires using HMMER v3.0 program (http://hmmer.org/, accessed on 19 November 2024) with “trusted cutoff” as the threshold. Retrieved proteins of each profile were selected to build a *S. bicolor*-specific LRR HMM profile using the “hmmbuild” module of the HMMER v3.0 package. By repeating the screening step, the output of hmmsearch was then manually verified using the SMART (http://smart.embl-heidelberg.de/, accessed on 19 November 2024) database and program TMHMM v2.0 [56] for transmembrane prediction. The final set of proteins were regarded as putative RLP proteins in *S. bicolor*.

A neighbor-jointing method based on 1000 bootstrap replicates was applied to generate a phylogenetic tree [57]. The evolutionary distance was computed using the Poisson correction method [58].

### 4.7. Cloning of SgR1 Promoter and Transformation

Approximately 1.9 kb of the *SgR1* promoter (*SgR1*p) were cloned using specific primers (SgR1_GUS), as shown in Supplementary Table S1. Amplified fragments were cloned into SalI-digested binary vector pBI101 (Clontech Laboratories, San Jose, CA, USA) using In-Fusion HD Cloning Kit (Clontech Laboratories, USA) according to the manufacturer’s instructions. Recombinant plasmid was introduced into *Escherichia coli* to enrich and was sequenced for correct clones. The plasmids with the correct *SgR1*p were transformed into *Agrobacterium tumefaciens* strain LBA4404 via freeze-thaw method. Transformation was into *Arabidopsis* (Col-0 Ecotype) plants by a floral dip method [59] and selected on a MS agar plate containing 50 mg^−1^ kanamycin.

### 4.8. Histochemical Analysis

Methods for histochemical assay was adopted from Franks’ lab at North Carolina State University. GPA-infested and non-infested leaves were excised and prefixed in ice-cold 90% Acetone followed by vacuum filtration. After subsequent vacuum filtration with a staining buffer (50 mM phosphate Buffer, 0.2% Triton X, 2 mM Ferro/Ferri) without X-Gluc, and with 2mM X-Gluc, samples were incubated at 37 °C for two days, and dehydrated by sequential changes of 20%, 35%, and 50% ethanol followed by FAA (50% Ethanol, 3.7% Formaldehyde, 5% acetic acid) incubation for 30 min. Samples were transferred to 70% ethanol and stored at 4 °C until examination under the Olympus BX51 microscope (Olympus Corporation, Tokyo, Japan).

### 4.9. Statistical Analysis

For the aphid count data, ANOVA and Tukey test was used to estimate the significant difference. For gene expression analysis, the relative expression level of each gene was calculated using the 2^−ΔΔCt^ method and the data presented are the averages of three biological and two technical replicates. Error bars represent standard error among replicates (n = 3) and the asterisks represent statistically significant changes between the controls and aphid infested samples as determined using Student’s *t*-test, * *p*  <  0.05, ** *p*  <  0.01, while the bars without asterisk are non-significant (*p* > 0.05).

## Data Availability

The data supporting the findings of this study are available in the Supplementary Materials of this article, so all data are presented in the paper.

## Supplementary Materials

The following supporting information can be downloaded at: https://www.mdpi.com/article/10.3390/ijms26010019/s1.

## Abbreviations

GBI	Greenbug [*Schizaphis graminum* (Rondani)] biotype I
GPA	Green peach aphids [*Myzus persicae* (Sülzer)]
LRR-RLP	Leucine-rich repeat containing receptor-like protein
NBS-LRR	Nucleotide binding site-leucine-rich repeat
SgR1	Schizaphis graminum resistance gene 1
SNP	Single-nucleotide polymorphism
QTLs	Quantitative trait loci
R genes	Resistance genes
RILs	Recombinant inbred lines
*q*RT-PCR	Quantitative real-time-polymerase chain reaction
InDel	Insertion/deletion

## References

[1] Gilding E.K., Frère C.H., Cruickshank A., Rada A.K., Prentis P.J., Mudge A.M., Mace E.S., Jordan D.R., Godwin I.D. (2013). Allelic Variation at a Single Gene Increases Food Value in a Drought-Tolerant Staple Cereal. Nat. Commun..

[2] Harvey T.L., Hackerott H.L. (1969). Recognition of a Greenbug Biotype Injurious to Sorghum1. J. Econ. Entomol..

[3] Harvey T.L., Kofoid K.D., Martin T.J., Sloderbeck P.E. (1991). A New Greenbug Virulent to E-Biotype Resistant Sorghum. Crop Sci..

[4] Harvey T.L., Wilde G.E., Kofoid K.D., Bramel-Cox P.J. (1994). Temperature Effects on Resistance to Greenbug (Homoptera: Aphididae) Biotype I in Sorghum. J. Econ. Entomol..

[5] Eddleman B.R., Chang C.C., McCarl B.A., Wiseman B.R., Webster J.A. (1999). Economic Benefits from Grain Sorghum Variety Improvement in the United States. Economic, Environmental, and Social Benefits of Resistance in Field Crops.

[6] Burd J.D., Porter D.R. (2006). Biotypic Diversity in Greenbug (Hemiptera: Aphididae): Characterizing New Virulence and Host Associations. J. Econ. Entomol..

[7] Huang Y. (2011). Improvement of Crop Protection against Greenbug Using the Worldwide Sorghum Germplasm Collection and Genomics-Based Approaches. Plant Genet. Resour..

[8] Kofoid K.D., Perumal R., Reese J.C., Campbell L.R. (2012). Registration of Twelve Sorghum Germplasm Lines Tolerant to Greenbug Feeding Damage. J. Plant Regist..

[9] Porter D.R., Burd J.D., Shufran K.A., Webster J.A., Teetes G.L. (1997). Greenbug (Homoptera: Aphididae) Biotypes: Selected by Resistant Cultivars or Preadapted Opportunists?. J. Econ. Entomol..

[10] Katsar C.S., Paterson A.H., Teetes G.L., Peterson G.C. (2002). Molecular Analysis of Sorghum Resistance to the Greenbug (Homoptera: Aphididae). J. Econ. Entomol..

[11] Agrama H., Widle G., Reese J., Campbell L., Tuinstra M. (2002). Genetic Mapping of QTLs Associated with Greenbug Resistance and Tolerance in *Sorghum bicolor*. Theor. Appl. Genet..

[12] Nagaraj N., Reese J.C., Tuinstra M.R., Smith C.M., Amand P.S., Kirkham M.B., Kofoid K.D., Campbell L.R., Wilde G.E. (2005). Molecular Mapping of Sorghum Genes Expressing Tolerance to Damage by Greenbug (Homoptera: Aphididae). J. Econ. Entomol..

[13] Wu Y., Huang Y. (2008). Molecular Mapping of QTLs for Resistance to the Greenbug *Schizaphis graminum* (Rondani) in *Sorghum bicolor* (Moench). Theor. Appl. Genet..

[14] Punnuri S., Huang Y., Steets J., Wu Y. (2013). Developing New Markers and QTL Mapping for Greenbug Resistance in Sorghum [*Sorghum bicolor* (L.) Moench]. Euphytica.

[15] Paterson A.H., Bowers J.E., Bruggmann R., Dubchak I., Grimwood J., Gundlach H., Haberer G., Hellsten U., Mitros T., Poliakov A. (2009). The *Sorghum bicolor* Genome and the Diversification of Grasses. Nature.

[16] McCormick R.F., Truong S.K., Sreedasyam A., Jenkins J., Shu S., Sims D., Kennedy M., Amirebrahimi M., Weers B.D., McKinley B. (2018). The *Sorghum bicolor* Reference Genome: Improved Assembly, Gene Annotations, a Transcriptome Atlas, and Signatures of Genome Organization. Plant J..

[17] Vos† P., Simons† G., Jesse T., Wijbrandi J., Heinen L., Hogers R., Frijters A., Groenendijk J., Diergaarde P., Reijans M. (1998). The Tomato Mi-1 Gene Confers Resistance to Both Root-Knot Nematodes and Potato Aphids. Nat. Biotechnol..

[18] Wroblewski T., Piskurewicz U., Tomczak A., Ochoa O., Michelmore R.W. (2007). Silencing of the Major Family of NBS–LRR-Encoding Genes in Lettuce Results in the Loss of Multiple Resistance Specificities. Plant J..

[19] Zhu-Salzman K., Salzman R.A., Ahn J.-E., Koiwa H. (2004). Transcriptional Regulation of Sorghum Defense Determinants against a Phloem-Feeding Aphid. Plant Physiol..

[20] Zhang H., Huang J., Huang Y. (2022). Identification and Characterization of Plant Resistance Genes (R Genes) in Sorghum and Their Involvement in Plant Defense against Aphids. Plant Growth Regul..

[21] Rooney H.C.E., van’t Klooster J.W., van der Hoorn R.A.L., Joosten M.H.A.J., Jones J.D.G., de Wit P.J.G.M. (2005). Cladosporium Avr2 Inhibits Tomato Rcr3 Protease Required for Cf-2-Dependent Disease Resistance. Science.

[22] Wulff B.B.H., Heese A., Tomlinson-Buhot L., Jones D.A., de la Peña M., Jones J.D.G. (2009). The Major Specificity-Determining Amino Acids of the Tomato Cf-9 Disease Resistance Protein Are at Hypervariable Solvent-Exposed Positions in the Central Leucine-Rich Repeats. Mol. Plant-Microbe Interact..

[23] Larkin M.A., Blackshields G., Brown N.P., Chenna R., McGettigan P.A., McWilliam H., Valentin F., Wallace I.M., Wilm A., Lopez R. (2007). Clustal W and Clustal X Version 2.0. Bioinformatics.

[24] Louis J., Gobbato E., Mondal H.A., Feys B.J., Parker J.E., Shah J. (2012). Discrimination of Arabidopsis PAD4 Activities in Defense against Green Peach Aphid and Pathogens. Plant Physiol..

[25] Puterka G.J., Peters D.C. (1995). Genetics of Greenbug (Homoptera: Aphididae) Virulence to Resistance in Sorghum. J. Econ. Entomol..

[26] Belfanti E., Silfverberg-Dilworth E., Tartarini S., Patocchi A., Barbieri M., Zhu J., Vinatzer B.A., Gianfranceschi L., Gessler C., Sansavini S. (2004). The HcrVf2 Gene from a Wild Apple Confers Scab Resistance to a Transgenic Cultivated Variety. Proc. Natl. Acad. Sci. USA.

[27] Larkan N.J., Lydiate D.J., Parkin I.a.P., Nelson M.N., Epp D.J., Cowling W.A., Rimmer S.R., Borhan M.H. (2013). The Rassica Napus Blackleg Resistance Gene Encodes a Receptor-like Protein Triggered by the Eptosphaeria Maculans Effector AVRLM1. New Phytol..

[28] Larkan N.J., Ma L., Borhan M.H. (2015). The Rassica Napus Receptor-like Protein RLM2 Is Encoded by a Second Allele of the Ep3/Lm2 Blackleg Resistance Locus. Plant Biotechnol. J..

[29] Park S.-J., Huang Y., Ayoubi P. (2006). Identification of Expression Profiles of Sorghum Genes in Response to Greenbug Phloem-Feeding Using cDNA Subtraction and Microarray Analysis. Planta.

[30] Punnuri S., Huang Y. (2017). Identification and Confirmation of Greenbug Resistance Loci in an Advanced Mapping Population of Sorghum. J. Agric. Sci..

[31] Huang Y., Huang J. (2023). Analysis of Plant Expression Profiles Revealed That Aphid Attack Triggered Dynamic Defense Responses in Sorghum Plant. Front. Genet..

[32] Abe H., Urao T., Ito T., Seki M., Shinozaki K., Yamaguchi-Shinozaki K. (2003). Arabidopsis AtMYC2 (bHLH) and AtMYB2 (MYB) Function as Transcriptional Activators in Abscisic Acid Signaling. Plant Cell.

[33] Eulgem T., Rushton P.J., Schmelzer E., Hahlbrock K., Somssich I.E. (1999). Early Nuclear Events in Plant Defence Signalling: Rapid Gene Activation by WRKY Transcription Factors. EMBO J..

[34] Hartmann U., Sagasser M., Mehrtens F., Stracke R., Weisshaar B. (2005). Differential Combinatorial Interactions of Cis-Acting Elements Recognized by R2R3-MYB, BZIP, and BHLH Factors Control Light-Responsive and Tissue-Specific Activation of Phenylpropanoid Biosynthesis Genes. Plant Mol. Biol..

[35] Urao T., Yamaguchi-Shinozaki K., Urao S., Shinozaki K. (1993). An Arabidopsis Myb Homolog Is Induced by Dehydration Stress and Its Gene Product Binds to the Conserved MYB Recognition Sequence. Plant Cell.

[36] Shrestha K., Huang J., Yan L., Doust A.N., Huang Y. (2024). Integrated Transcriptomic and Pathway Analyses of Sorghum Plants Revealed the Molecular Mechanisms of Host Defense against Aphids. Front. Plant Sci..

[37] Baker S.S., Wilhelm K.S., Thomashow M.F. (1994). The 5′-Region of Arabidopsis Thaliana Cor15a Has Cis-Acting Elements That Confer Cold-, Drought- and ABA-Regulated Gene Expression. Plant Mol. Biol..

[38] Jiang C., Iu B., Singh J. (1996). Requirement of a CCGAC Cis-Acting Element for Cold Induction of the BN115 Gene from Winter Brassica Napus. Plant Mol. Biol..

[39] Elmore J.M., Lin Z.-J.D., Coaker G. (2011). Plant NB-LRR Signaling: Upstreams and Downstreams. Curr. Opin. Plant Biol..

[40] Botos I., Segal D.M., Davies D.R. (2011). The Structural Biology of Toll-like Receptors. Structure.

[41] Du B., Zhang W., Liu B., Hu J., Wei Z., Shi Z., He R., Zhu L., Chen R., Han B. (2009). Identification and Characterization of Bph14, a Gene Conferring Resistance to Brown Planthopper in Rice. Proc. Natl. Acad. Sci. USA.

[42] Tamura Y., Hattori M., Yoshioka H., Yoshioka M., Takahashi A., Wu J., Sentoku N., Yasui H. (2014). Map-Based Cloning and Characterization of a Brown Planthopper Resistance Gene BPH26 from *Oryza sativa* L. ssp. Indica Cultivar ADR52. Sci. Rep..

[43] Tuinstra M.R., Wilde G.E., Kriegshauser T. (2001). Genetic Analysis of Biotype I Greenbug Resistance in Sorghum. Euphytica.

[44] Van der Hoorn R.A.L., Roth R., De Wit P.J.G.M. (2001). Identification of Distinct Specificity Determinants in Resistance Protein Cf-4 Allows Construction of a Cf-9 Mutant That Confers Recognition of Avirulence Protein AVR4. Plant Cell.

[45] van der Hoorn R.A.L., Wulff B.B.H., Rivas S., Durrant M.C., van der Ploeg A., de Wit P.J.G.M., Jones J.D.G. (2005). Structure-function analysis of cf-9, a receptor-like protein with extracytoplasmic leucine-rich repeats. Plant Cell.

[46] Li J., Brader G., Palva E.T. (2004). The WRKY70 Transcription Factor: A Node of Convergence for Jasmonate-Mediated and Salicylate-Mediated Signals in Plant Defense. Plant Cell.

[47] Miao Y., Zentgraf U. (2007). The Antagonist Function of Arabidopsis WRKY53 and ESR/ESP in Leaf Senescence Is Modulated by the Jasmonic and Salicylic Acid Equilibrium. Plant Cell.

[48] Xu X., Chen C., Fan B., Chen Z. (2006). Physical and Functional Interactions between Pathogen-Induced Arabidopsis WRKY18, WRKY40, and WRKY60 Transcription Factors. Plant Cell.

[49] Mauch-Mani B., Mauch F. (2005). The Role of Abscisic Acid in Plant–Pathogen Interactions. Curr. Opin. Plant Biol..

[50] Oide S., Bejai S., Staal J., Guan N., Kaliff M., Dixelius C. (2013). A Novel Role of PR2 in Abscisic Acid (ABA) Mediated, Pathogen-Induced Callose Deposition in Arabidopsis Thaliana. New Phytol..

[51] Nicholson S.J., Puterka G.J. (2014). Variation in the Salivary Proteomes of Differentially Virulent Greenbug (*Schizaphis graminum* Rondani) Biotypes. J. Proteom..

[52] Rosentrater K.A., Evers A.D. (2018). Kent’s Technology of Cereals.

[53] Webster F.M., Phillips W.J. (1912). The Spring Grain-Aphis or “Green Bug”.

[54] Wu Y., Huang Y., Tauer C., Porter D.R. (2006). Genetic Diversity of Sorghum Accessions Resistant to Greenbugs as Assessed with AFLP Markers. Genome.

[55] Murray M.G., Thompson W.F. (1980). Rapid Isolation of High Molecular Weight Plant DNA. Nucleic Acids Res..

[56] Krogh A., Larsson B., von Heijne G., Sonnhammer E.L.L. (2001). Predicting Transmembrane Protein Topology with a Hidden Markov Model: Application to Complete Genomes1. J. Mol. Biol..

[57] Saitou N., Nei M. (1987). The Neighbor-Joining Method: A New Method for Reconstructing Phylogenetic Trees. Mol. Biol. Evol..

[58] Zuckerkandl E., Pauling L., Bryson V., Vogel H.J. (1965). Evolutionary Divergence and Convergence in Proteins. Evolving Genes and Proteins.

[59] Clough S.J., Bent A.F. (1998). Floral Dip: A simplified method for Agrobacterium-mediated transformation of *Arabidopsis thaliana*. Plant J..

