# Supplementary material for: SgR1, Encoding a Leucine-Rich Repeat Containing Receptor-like Protein, Is a Major Aphid (Schizaphis graminum) Resistance Gene in Sorghum
Source: Int J Mol Sci. 2024 Dec 24;26(1):19. doi: 10.3390/ijms26010019 (PMC11719657; doi:10.3390/ijms26010019)
Supplement: Supplementary file 1 [file ijms-26-00019-s001.zip › ijms-3353601-supplementary.pdf]

Supplementary Materials:

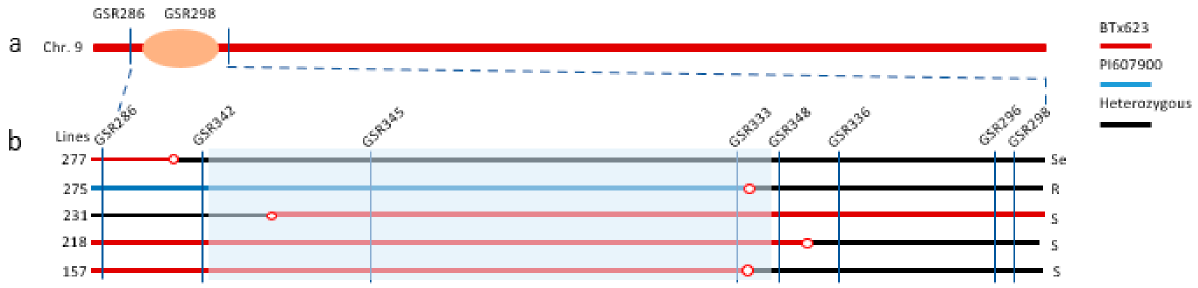

Figure S1. (**a**) Location of *SgR1* on chromosome 9. (**b**) Genotypes and phenotypes of five recombinant lines. Shaded area indicates the fine mapped SgR1 region flanking with DNA markers GSR342 and GSR348.

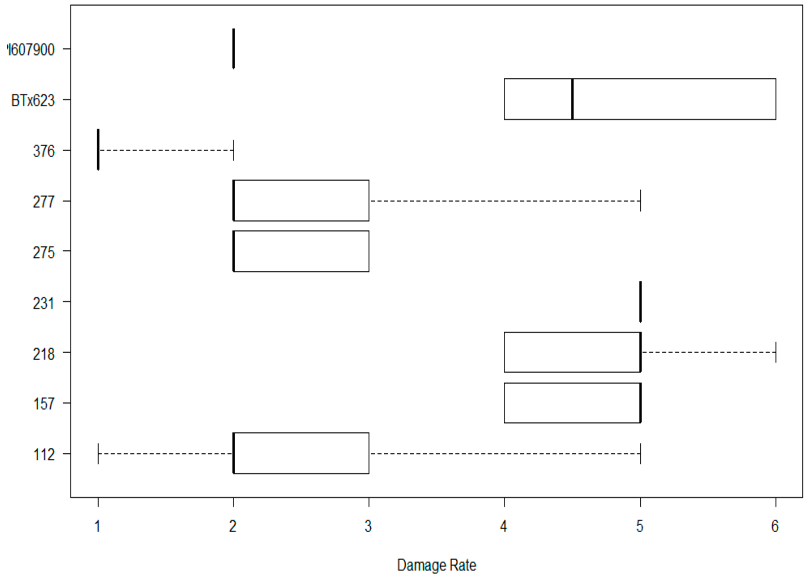

Figure S2. Phenotypes of parents and recombinants' progenies illustrated by box plots, showing plant damage caused by greenbugs.

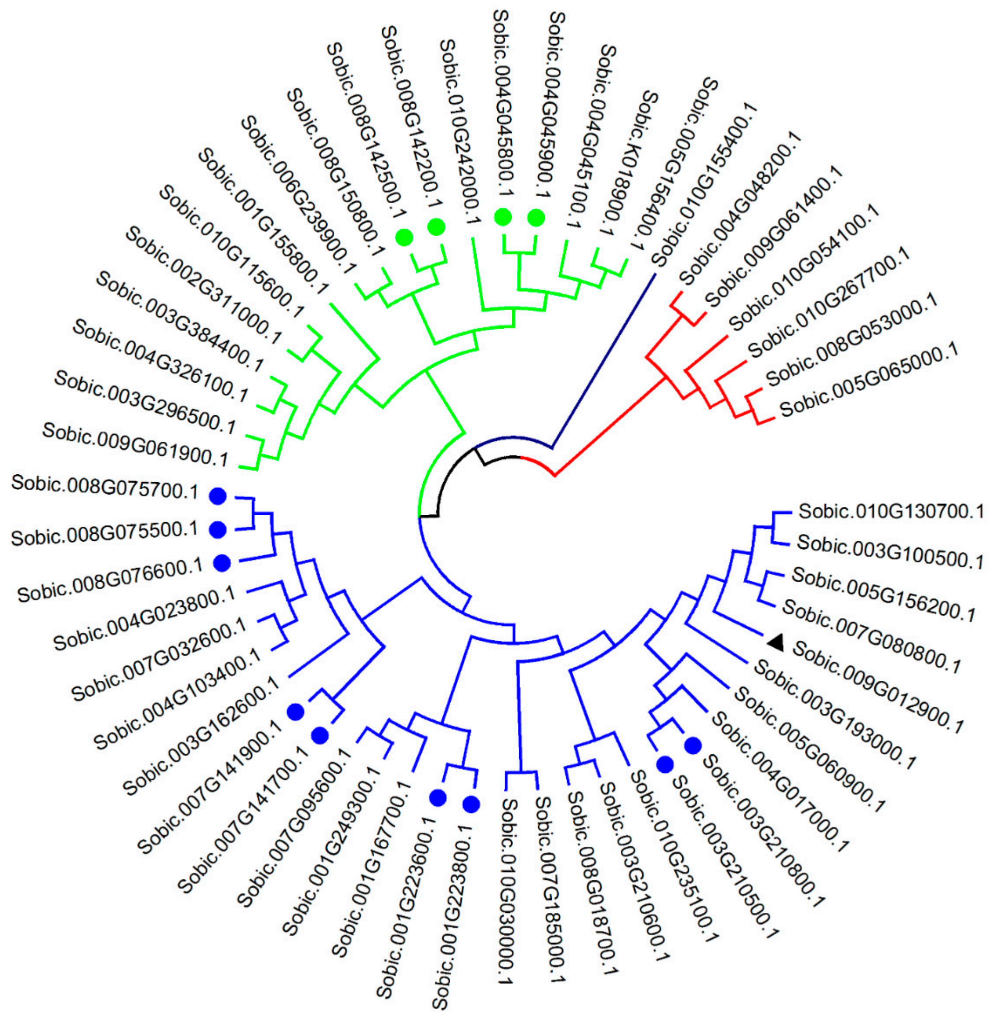

Figure S3. An unrooted phylogenetic tree constructed using all putative RLP proteins from *S. bicolor*.

Table S1. List of primers used in this study.

Primer name	Forward	Reverse
GSR286	GGTGGCCACTGTCTTCTTGT	CAGCAGCATCTGGTTGAAGA
GSR342	CGCAGGTGAGGATCGATTA	CATGTGCTCATGTGCTGTGA
GSR345	TCACTCGTCAGCCACAACTC	GAAGTATTCGTCCCCTTCCA
SP49	TGTCACCAGTAAGCCGCTTTG	GCCAATCAGGAGATGTGGTC
GSR383	ATTCCACAGCCCTTCACATC	GAAGTGGCACATGTGGTTTG
GSR353	ATGGAATTTCGGTGCTTCAG	ATCCACCACCCAACTCAGTC
GSR376	CTGTTTGCAGGGGATGTTTC	CTAACTCCTGCGCCTAAACC
GSR333	TGTTCATCTCCAACGTCTCG	GCAGCATCTCGTCAGAATCA
GSR348	GCGGATTTTTCCTTGATGTG	CTTGGCATTTCTTGTCACCA
GSR336	AGATGAGGCAGAAACCGAAG	GACAACAGGCTTCTCCTTGC
GSR296	GATGTTCGACTCCCTGCAC	CCGTTCTCCAGCAGCACCT
GSR298	TGCCTCCTCTTCCTCTTCCT	AGGACCATGGACAGCACCT
SgR1_qPCR	ATGTGCCTCCTGCTCTTT	AGTAACGCCTCCTTCTCG
SgR1_promoter	GATCTCTTGCCCATCCACTCT	CAGCTGCCATTGAACTTCTTC
SgR1_CDS	CACCGACTGCAAACAGAAGA	GACCTTGTTTGGATGTGGTT
SgR1_GUS	CGACTCTAGAGGATCCGTTTTCGTCGCATGCCTTA	ACCTACCCGGGGATCCGTCGGTGCTTGATGGAAAA
